# Digital technology-based dyadic interventions in patients with mild cognitive impairment or dementia and their caregivers: a scoping review

**DOI:** 10.3389/fpsyt.2026.1726605

**Published:** 2026-03-12

**Authors:** Mengyao Wang, Yue He, Jinling Song, Hang Li, Nannan Geng, Changying Li, Zhenzhu Jiao, Yu Gao, Yang Wang

**Affiliations:** 1 School of Nursing, Changchun University of Chinese Medicine, Changchun, Jilin, China; 2 The Third Affiliated Hospital of Changchun University of Chinese Medicine, Changchun Jilin, China

**Keywords:** dementia, digital technology, dyadic interventions, informal caregivers, mild cognitive impairment, scoping review

## Abstract

**Background:**

Cognitive impairment, particularly when progressing to dementia, exerts considerable psychological distress on both patients and their caregivers. Dyadic interventions regard patients and caregivers as a single unit, emphasizing their interdependence in disease management and highlighting its critical relevance to health outcomes. In recent years, digital technology has been increasingly incorporated into dyadic interventions to support individuals with cognitive impairment and their caregivers. While relevant scoping reviews have been conducted in the cancer field, systematic syntheses of digital dyadic interventions for geriatric cognitive impairment remain limited.

**Aim:**

This study conducts a scoping review to evaluate the use of digital dyadic interventions for individuals with mild cognitive impairment or dementia and their caregivers. It summarizes key implementation features across intervention types and technologies and identifies major barriers and optimization strategies for wider adoption in home and community care.

**Methods:**

Publications involving digital technology–based dyadic interventions targeting individuals with cognitive impairment and their caregivers were included in the review. Searches were conducted across five databases: PubMed, Embase, EBSCOhost, Web of Science, and IEEE Xplore. Search terms included digital-related keywords such as “online,” “remote,” “digital,” and “virtual,” combined with terms including “dyadic interventions,” “dyadic coping,” “cognitive impairment,” and “cognitive disorders.” A narrative synthesis approach was employed to analyze the retrieved literature, focusing on participant characteristics, intervention methods, implementation contexts, and reported outcomes. These elements were subsequently synthesized.

**Results:**

A total of 38 studies from various countries were included, involving individuals with mild cognitive impairment or dementia and their caregivers. Interventions were classified into four categories: mobile applications, immersive sensory systems, remote platforms, and intelligent interactive agents. Most studies reported improvements in patients’ cognition, emotional well-being, and quality of life, alongside reduced caregiver burden and enhanced relationship quality. Barriers included limited technology access, uneven digital literacy, and privacy and security concerns.

**Conclusion:**

Dyadic digital interventions can enhance the quality of life and relationship interactions for both patients and caregivers. Their effectiveness relies on collaborative participation but is constrained by technological accessibility and usage capabilities. Future efforts should optimize design and implementation to promote their sustainable application in care settings.

**Systematic review registration:**

DOI: 10.17605/OSF.IO/TRGQ5

## 1 Introduction

With the accelerating aging of the population, mild cognitive impairment (MCI) and dementia have emerged as major global public health challenges (1, 2). MCI represents a transitional stage between normal cognition and dementia (2, 3). Its prevalence increases non-linearly with age: 8.4% among those aged 65–69, 10.1% aged 70–74, 14.8% aged 75–79, and 25.2% aged 80–84 (4), and is associated with an elevated risk of progression to dementia (5). Dementia is characterized by cognitive impairment that severely compromises social and occupational functioning. The global number of individuals living with dementia is projected to reach 152 million by 2050, with approximately 68% residing in low- and middle-income countries (2, 6, 7).

Individuals with cognitive impairment typically require long-term care provided by family caregivers (8). These caregivers play a critical role in disease management but frequently face substantial economic, physical, and emotional burdens (9). Owing to patients’ cognitive and communication limitations, caregivers often experience physical discomfort in addition to anxiety and depression (10, 11). Dyadic interventions regard patients and caregivers as joint participants within a unified care plan, thereby improving health outcomes for both simultaneously (12–14). Digital health technologies generate and apply health data through electronic platforms, software, and hardware (15, 16), thereby overcoming geographic and economic barriers and enhancing the accessibility and reach of dyadic interventions. Existing research indicates (17, 18) that digital dyadic interventions can help maintain patients’ cognitive and psychological states, enhance family interactions, and strengthen caregivers’ skills and perceived support. Across the continuum from mild cognitive impairment to severe dementia, the format of digital dyadic interventions varies according to disease stage. Beentjes et al. (18) used the tablet-based “FindMyApps” tool to help individuals with MCI or mild dementia and their caregivers identify dementia-friendly applications, thereby improving self-management and social participation. Laver et al. (49) delivered tablet-based videoconferencing interventions in which occupational therapists provided environmental assessment, problem-solving strategies, and skills training for dyads with mild-to-moderate dementia to strengthen caregiver confidence and slow functional decline. Rochon et al. (11) employed a web-based low-immersion virtual environment integrating natural soundscapes and multisensory activities for dyads that included individuals with moderate-to-severe dementia, aiming to reduce caregiver stress and enhance real-time engagement and emotional connectedness. However, existing research predominantly focuses on the effects of dual interventions for cancer patients and caregivers, with limited systematic reviews of digital dual interventions for individuals with MCI or dementia and their caregivers. Particularly, there is a lack of summarization regarding intervention formats, mechanisms of action, and implementation challenges.

Therefore, this study is designed to conduct a scoping review that summarizes digital technology–based dyadic interventions for patients with MCI and dementia, as well as their caregivers. It will organize the main types, technological platforms, mechanisms of action, and the effectiveness and barriers encountered during the implementation of existing digital dyadic interventions. From a dual-interaction perspective, this study describes the characteristics of different intervention formats and analyzes their potential impacts and challenges for patients and caregivers. This approach aims to provide a clearer understanding of various digital dual intervention strategies and their current application status for individuals with cognitive impairment and their caregivers.

## 2 Materials and methods

### 2.1 Design

This study employed the five-phase framework for scoping reviews proposed by Arksey and O’Malley (19), sequentially comprising (1): defining research objectives (2); retrieving relevant literature (3); screening studies (4); Data extraction (5); Synthesis, summary, and reporting of findings. The review centers on core issues, including primary types and technological forms of digital dyadic interventions, their mechanisms of action, and outcomes and barriers encountered during implementation. A scoping review systematically maps the breadth and diversity of existing literature, providing a more comprehensive overview of the current state of rehabilitation interventions using digital Dyadic interventions for individuals with mild cognitive impairment or dementia and their caregivers. Accordingly, the core questions addressed in this study are: What specific forms do current digital dual interventions for individuals with cognitive impairment and their caregivers take? How are they implemented? What are the facilitating and impeding factors? What are the primary existing challenges? This review has been registered on the Open Science Framework website: https://osf.io/ftnp2.

### 2.2 Search strategy and eligibility criteria

**Figure 1:**
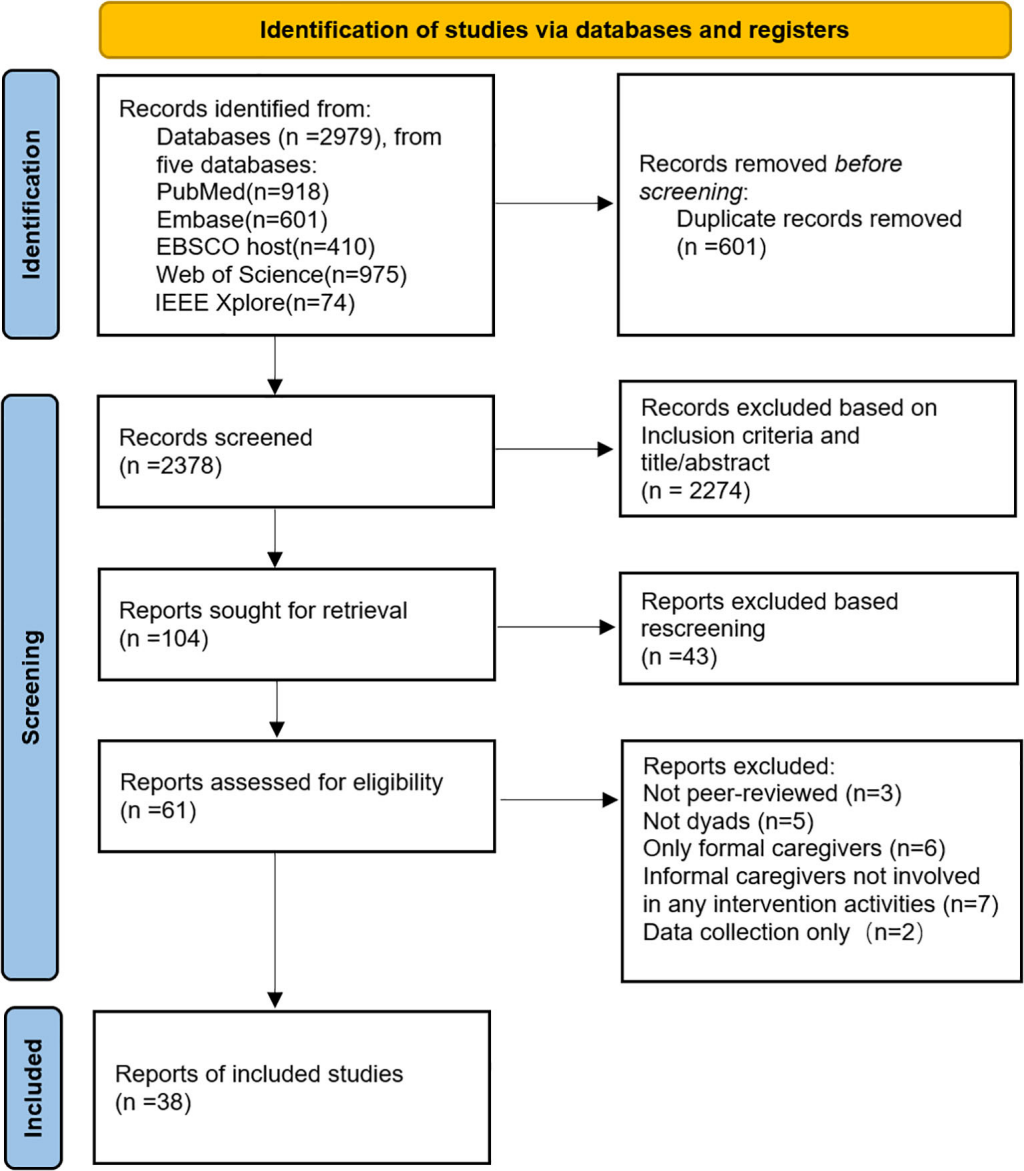
PRISMA flowchart.

A comprehensive literature search was conducted using five major databases: Scopus, PubMed, Web of Science, Embase, EBSCOhost, and IEEE Xplore. Grey literature was identified using the Google Scholar search engine. These databases broadly cover relevant literature in mental health, medicine, and nursing. The search terms used are detailed in **Supplementary Table 2**. The article selection process was documented using the Preferred Reporting Items for Systematic Reviews and Meta-Analyses (PRISMA) flow diagram (20): (**Figure 1**).

### 2.3 Inclusion and exclusion criteria

The PCC (Population, Concept, Context) framework guided inclusion criteria (21). Population: Pairs where one member has been diagnosed with dementia of any type or severity or MCI, and the other is their family caregiver. Concept: Interventions delivered in home or community settings using digital technologies—including but not limited to apps, online platforms, remote monitoring, and virtual reality—to both MCI/dementia patients and their caregivers. Context: Interventions must be delivered in home or community settings, either remotely by researchers or self-administered by participants with caregiver assistance. Accordingly, the literature inclusion criteria are (1): Study subjects comprise dyads of MCI/dementia patients and caregivers, with caregivers primarily informal (2); Interventions targeting both patients and caregivers with explicit bidirectional effects or collaborative objectives (3); Interventions delivered via digital technology (4); Reporting at least one health outcome or intervention effect relevant to patients, caregivers, or both.

The following studies will be excluded (1): interventions that target only patients or only caregivers, rather than both (2); interventions or evaluations that are not based on digital technology (3); studies involving exclusively formal caregivers, thereby excluding informal caregivers (4); interventions where informal caregivers did not actively participate in any interactive components (5); secondary literature, including systematic reviews, editorials, commentaries, and narrative reviews, as well as studies without available full texts. This study includes original research providing primary empirical data, as well as research protocols that clearly describe intervention design, participant structure, and implementation processes, thereby ensuring that the results focus on intervention strategies, research methods, and population characteristics, while avoiding redundant summaries of secondary.

### 2.4 Data extraction

Data extraction was performed in Microsoft Excel 2020. Fields included basic study information (authors, publication year, country), study design, sample characteristics (patients and their caregivers), intervention type, form of digital technology, implementation method, intervention duration, outcome measures, and primary outcomes. A descriptive summary table was constructed based on these data. Data extraction was performed independently by two researchers, W.MY and H.Y. Disagreements were resolved through discussion, with final decisions made by a third researcher, W.Y. The search period was set from January 2000 to July 2025. References from included studies were traced to identify potential additional studies. All records were imported into EndNote X9, deduplicated, and then subjected to the next screening step.

### 2.5 Quality appraisal

The Mixed-Methods Research Quality Assessment Tool (MMAT, 2018 version) was used to evaluate the quality of included studies (22). The MMAT establishes five methodological criteria for each of five study designs: qualitative research, randomized controlled trials, non-randomized quantitative research, quantitative descriptive research, and mixed-methods research. Response options for each criterion include “Yes,” “No,” and “Can not tell.” Subsequently, the overall quality score was calculated as the percentage of criteria successfully met, assigning studies to one of four quality grades: Grade A (indicating 80–100% compliance), Grade B (60–79% compliance), Grade C (40–59% compliance), and Grade D (below 40% compliance). Quality assessments were conducted independently by W.MY and H.Y. Discrepancies were resolved through discussion; unresolved disagreements were adjudicated by a third researcher, W.Y. To ensure methodological rigor and transparency, the five-step framework proposed by Arksey and O’Malley was strictly followed, and a systematic analysis of the current research status was conducted based on key elements, including intervention formats, participant characteristics, and intervention outcomes.

### 2.6 Analysis

A descriptive analysis approach was employed to systematically categorize and synthesize the included literature. Two researchers with expertise in the field independently reviewed the full texts and performed open coding to extract quantitative data on intervention duration and key outcomes, as well as qualitative information on intervention experiences, implementation barriers, and facilitating factors. Subsequently, inductive thematic analysis was applied to consolidate similar concepts into overarching themes, which were then organized and interpreted in relation to intervention objectives, content components, collaborative participation methods, technology types, and implementation challenges. Disagreements were resolved through discussion, and adjudication by a third researcher was sought when necessary. Ultimately, the findings were presented in tables, figures, and narrative text to illustrate the typological characteristics, participant demographics, and effectiveness of digital dyadic interventions, thereby providing a reference for the design and optimization of future intervention strategies.

## 3 Results

### 3.1 Selection and inclusion of studies

Initial searches across five databases and Google Scholar identified 2,979 records. After deduplication in EndNote X9, 601 duplicate records were removed, leaving 2,378 unique records. Based on the inclusion criteria, a preliminary screening of titles and abstracts retained 104 articles. A total of 2,274 records were excluded: 714 for not involving individuals with dementia or their caregivers; 203 for not being related to digital technology; and 1,357 for not addressing dyadic interventions targeting both patients and caregivers. Subsequent review of titles and abstracts of the 104 articles resulted in the exclusion of 43 articles: 23 because the participants were exclusively formal caregivers, and 20 because the intervention or evaluation was not based on digital technology, yielding 61 articles. After full-text assessment,23 records were excluded: 3 were not published in peer-reviewed journals;5 did not target both patients and caregivers; 6 included only formal caregivers; 7 for interventions where informal caregivers did not engage in interactive components, and 2 for studies collecting data without assessment or intervention Ultimately, 38 studies met the eligibility criteria and were included in the final analysis.

### 3.2 Study characteristics

**Table 1** provides an overview of the included articles. These 38 studies were conducted across 12 countries: 13 from the United States (11, 23–34), 2 from Canada (35, 36), 3 from the Netherlands (17, 18, 37), 7 from the United Kingdom (38–44), 1 from Norway (45), 4 from Australia (46–49), 2 from China (50, 51), 1 from Belgium (52), 1 from Germany (53), 2 from Italy (54, 55), and 2 multinational studies (56, 57). 4 were non-randomized studies (28, 51, 54, 55), 11 were mixed studies (11, 30, 33, 34, 36, 42, 44, 45, 48, 52, 53), 7 were qualitative studies (23, 24, 35, 40, 43, 46, 57), 11 were RCTs (17, 18, 25, 27, 29, 32, 37–39, 47, 49), 2 were quantitative descriptive studies (41, 56), 1 was a randomized controlled trial protocol (50), and 2 were mixed-methods research protocols (26, 31). Further details of the included studies are provided in **Supplementary Table 1**.

**Table 1:** Information of the studies that were included.

Studies (Lead author, year)	Country	Study design/type^a^	Participants/population of interest	Technology	Synergistic participation (Yes or no)	Type of caregiver	Duration (months/weeks)	Outcomemeasures for patients^c^	Outcome measures for carers^c^
Afifi et al. (2023) (28)	USA	Non-randomized study	21 dyads; MCI/mild-moderate dementia	Rendever VR platform	Yes	Informal caregivers (family members, primarily adult children)	3 weeks	• QOL-AD: • MHI-5 • GDS-SF • PSS • PANAS-SF	• Caregiver Burden Index • CES-D • Mental Health Inventory-5 •PSS •PANAS-SF •URCS •RSS
Rochon et al. (2025) (11)	USA	Mixed study	9 dyads; Dementia	Immersive Virtual Environment Technology	Yes	Informal caregivers (spouse, adult child)	N/A^b^	• Feasibility questions • SUS	• SUS • Feasibility questions
Hastings et al. (2021) (32)	USA	RCT	40 dyads; MCI	Video Connect on iPad	Yes	Informal caregivers (spouse or significant other)	12 weeks	•PASE •PHQ-9: •GAD-7 •MOS-SS •PROMIS-29 •PSQI •Perceived Value interviews	•SUS •Perceived Value interviews
Kuzmik, (2025) (24)	USA	Qualitative study	14 dyads; mild-moderate dementia	Mobile application	Yes	Informal caregivers (spouses)	N/A	Thematic Analysis	Thematic Analysis
Zubatiy et al. (2021) (33)	USA	Mixed study	10 dyads; MCI	Smart speaker device	Yes	Informal caregivers (spouses, one adult daughter)	10 weeks	•Logged Interactions independence •Thematic Analysis •Preference and Priority Survey	•Logged Interactions Independence •Thematic Analysis •Preference and Priority Survey
Rhodus et al. (2023) (29)	USA	RCT	28 dyads; Alzheimer’s disease	Video conferencing technology	Yes	Informal caregivers (family members/primary caregivers)	6 weeks	•Feasibility •COPM •MoCA	•Feasibility •NPI–Q, •ZBI •COPM
Song, (2024) (25)	USA	RCT	30 dyads; dementia	Video telehealth platform	Yes	Informal caregivers (spouses, daughters, sons, granddaughters)	5 weeks	•Sleep efficiency •Total wake time measured by actigraphy	•Sleep efficiency •Total wake time by actigraphy, •PSQI •ZBI •Positive Aspects of Caregiving
Peterson et al. (2020) (34)	USA	Mixed study	34 dyads; Alzheimer’s Disease and Related Dementias	Web-based Personal Health Record System	Yes	Informal caregivers (spouses, other family members)	12 months	•PHR-ADRD Feasibility and Utility Checklist •Monthly Log/Usage Data	•PHR-ADRD Feasibility and Utility Checklist •Monthly Log/Usage Data, •ZBI •CES-D
Peterson, (2023) (30)	USA	Mixed study	16 care partners + 11 drivers with memory loss	video conferencing technology	Yes	Informal caregivers (spouses)	3 months	•CarFreeMe™-Dementia Intervention Review Checklists •AMRT •Mobility Confidence Questionnaire	•CarFreeMe™-Dementia Intervention Review Checklists •AMRT •Mobility Confidence Questionnaire
Bannon, (2025) (23)	USA	Qualitative study	16 dyads; dementia	Video conferencing platform, Virtual interview technology	Yes	Informal caregivers (partners/spouses)	N/A	•Thematic Analysis	•Thematic Analysis
Rodriguez, (2023) (27)	USA	RCT	53 dyads; with Alzheimer’s Disease and Related Dementias	mobile telehealth application	Yes	Informal caregivers (family/friends)	6 months	•NPI	•SUS •BIQ •NPI-Caregiver Distress
Bannon et al. (2023) (31)	USA	Mixed study Protocol	20 dyads; Alzheimer’s Disease	Live Video Telehealth + Zoom	Yes (collaborative management of progressive symptoms)	Informal caregivers (spousal care-partners)	N/A	•HADS •GDS-Short Form •WHOQOL-BRIEF •Couple Satisfaction Index	•HADS •WHOQOL-BRIEF •CSI •PCS
Rochon et al. (2023) (26)	USA	Mixed study Protocol	11 dyads; dementia	Web-based platform	Yes (participates together)	Informal caregivers (family member or friend)	N/A	•VC-IOE •Adapted Feasibility Scale •SUS	•Adapted Feasibility Scale •SUS
Appel et al. (2023) (36)	Canada	Mixed study	7 dyads; mild-severe dementia	VR headset + tablet	Yes	Informal caregivers (spouse and child-parent pairs)	4 weeks	• QoL-AD • WHO-5 • ObsRVR	• QoL-AD • WHO-5 • SUS • Qualitative Observations
Valdivia & Li (2025) (35),	Canada	Qualitative study	17 elders + 16 family caregivers	Voice-reminders app	Not	Informal caregivers (Family caregivers)+Medical Care Providers	N/A	Qualitative Content Analysis	Qualitative Content Analysis
Beentjes, (2020) (18)	Netherlands	RCT	59 dyads; mild dementia/MCI	web-based selection tool and errorless learning training program	Yes	Informal caregivers (partners, children) and professional caregivers	3 months	•SMAS-S •ASCOT •MSPP	•PES •SSCQ •TOPICS-MDS
Dröes et al. (2019) (37)	Netherlands	RCT	189 dyads; mild-to-moderate dementia	telephone, web-based learning	Yes	Formal caregivers (professional caregivers) and informal caregivers (family members, relatives)	6 months	•NPI symptom severity: •Self-esteem scale •NPI	•SSCQ •NPI perceived burden
Elfrink, (2021) (17)	Netherlands	RCT	42 dyads; mild dementia	Multimedia timeline technology, Online reminiscence tools	Yes	Informal caregivers (spouses, family members)	6 months	•NPI	•EDIZ •NPI distress scales •TOPICS-MDS (self-rated distress) •CarerQol •RAND-36/Cantril’s Ladder
Tyack et al., (2017) (44)	UK	Mixed study	12 dyads; dementia	tablet with custom art-viewing app	Yes	Informal caregivers (family/friends)	2 weeks	• VAS composite well-being Score •QoL-AD	•QoL-AD • VAS composite well-being Score
Cooper et al. (2024) (39)	UK	RCT	302 dyads; dementia	Video-call/Telephone	Yes	Informal caregivers (Spouse/partner, Child, Friend, Other)	12 months	• GAS • DEMQOL/DEMQOL-proxy • DAD • NPI	• GAS • CarerQol •HADS •MCTS
Gonzalez, (2025) (38)	UK	RCT	377 dyads; dementia and sleep disturbance	Actigraphy watches , Video calling	Yes	Informal caregivers (spouses/partners, children)	8 months	•EQ-5D-5L proxy •SDI • QALYs	• SDI
Fowler-Davis (2020) (42),	UK	Mixed study	30 dyads; mild dementia	Internet of Things monitoring device, Smart home sensor technology	Yes	Informal caregivers (spouses/partners, children)	4 months	•EFS •WEMWBS (short form)	•ZBI •WEMWBS (short form):
Howe et al., (2020) (41)	UK	Quantitative descriptive study	37 dyads; mild to moderate dementia/cognitive impairment	A socially-enabled digital content distribution platform	Yes	Informal caregivers (spouses, children, friends)	6 months	•Logging Data (Platform engagement, informational, etc.) •Usefulness and Usability Questionnaire	•Logging Data (Platform engagement, informational, etc.) •Usefulness and Usability Questionnaire
Killin, (2018) (43)	UK	Qualitative study	10 dyads; Alzheimer’s, vascular, or mixed dementia	Integrated Multi-Component Support System	Yes	Informal caregivers (primarily spouses)	2 months	•NPT Constructs	•NPT Constructs
Wolverson et al., (2022) (40)	UK	Qualitative study	22 dyads; dementia/MCI	Caregivers pro-MMD website platform via tablets	Yes	Informal caregivers (spouses/partners, adult children, friends)	N/A	•Thematic Analysis	•Thematic Analysis
Puaschitz et al. (2023) (45)	Norway	Mixed study	82 dyads; dementia	active sensor	Not	Informal caregivers (children, spouses)	24 months	•SA access •SA Experiences	•SA access •SA Experiences
Laver, (2020) (49)	Australia	RCT	63 dyads; dementia and their	Multi-device Video Conferencing Platform	Yes	Informal caregivers (spouses, children, others)	4 months	•CAFU •Caregiver Behavioral Occurrence and Upset Scale	•CMI •Perceived Change Scale
Lang et al., (2023) (47)	Australia	RCT	9 dyads; dementia	Tablet-delivered Mindfulness and Video Communication System	Yes	Informal caregivers (spouses/partners)	3 months	•SPPB	•ZBI •SMS
Muñoz et al., (2021) (48)	Australia	Mixed Study	21 dyads; moderate to advanced dementia	tablet app with 8 games	Yes	Informal caregivers (partners, children) and care staff	3 months	• Data Logging (Game preference identification, engagement patterns, social interaction facilitation)	• Logging Data (Game preference identification, engagement patterns, social interaction facilitation)
Clark et al., (2024) (46)	Australia	Qualitative study	9 people with dementia + 6 care partners	Video conferencing platforms	Yes	Informal caregivers (spouses/family carers) and formal carers	10 weeks	•Thematic Analysis	•Thematic Analysis
Lai, (2020) (51)	China	Non-randomized study	60 dyads; Neurocognitive Disorder	Video apps, telephone calls	Yes	Informal caregivers (Spousal, caregivers)	1 month	•MoCA, •RMBPC •QoL-AD	•SF-36v2 •ZBI •RCSES
Wan Y, (2023) (50)	China	RCT protocol	358 dyads; dementia	Mobile application	Yes	Informal caregivers (family members living with patients)	18 months	•QOL-AD • Cornell Scale for Depression in Dementia • IADL	•ZBI Reduction •GAD-7
Monnet, (2024) (52)	Belgium	Mixed study	21 individuals with mild-moderate dementia + 31 family caregivers	Web-based Interactive Card Platform	Yes	Informal caregivers (partners, adult children)	2 months	•Logging Data (User engagement , perceived usefulness, barriers to use)	•Logging Data (User engagement , perceived usefulness, barriers to use)
Hoel et al., (2022) (53)	Germany	Mixed study	9 dyads; dementia	I-CARE tablet-based activation	Yes	Informal caregivers (spouses, siblings)	1 month	•QCPR •DEMQOL •FAST	•Carer-Qol-7D •BSFC (caregiver burden) •QCPR (relationship quality)
Stara et al. (2021) (55)	Italy	Non-randomized study	mild dementia (n=20) + Family caregivers (n=14)	Embodied Conversational Agent	Yes	Informal caregivers (spouses and sons)	4 weeks	•SUS score •Almere model •QOL-A	• SUS score •Almere model •QOL-AD
Amabili, (2022) (54)	Italy	Non-randomized study	9 dyads; dementia	Integrated IoT & Social Robotics Ecosystem	Yes	Informal caregivers (family members)	6 months	• Goal Attainment Scale • EQ-5D-5L:	• ZBI •EQ-5D-5L •UTAUT scale • SUS
Guzman-Parra et al. (2020) (56)	Spain & Sweden	Quantitative descriptive study	1086 dyads; MCI/early-stage dementia	Smartphones, tablets	Not	Informal caregivers (relationship not specified in detail)	N/A	•TechPH •Use and familiarity questionnaire •QoL-AD	•TechPH • ZBI-12 •EQ5D •Use and familiarity questionnaire
Notley, (2025) (57)	Australia & USA	Qualitative study	17 dyads; dementia	Integrated Mobile/IoT & Assistive Devices	Yes	Informal caregivers (co-resident, other)	N/A	•Thematic Analysis/Interpretive Description	•Thematic Analysis/Interpretive Description

a Non-randomized study examines intervention effects without random assignment, observing outcomes in naturally formed groups. Mixed studies integrate qualitative and quantitative approaches to capture both measurable outcomes and experiential insights. Randomized controlled trials randomly allocate participants to intervention or control groups to minimize bias and establish causal effects. Qualitative studies use interviews, focus groups, or observations to explore participants’ perceptions and experiences. Quantitative descriptive studies collect numerical data to describe population characteristics or patterns without testing causal relationships.

b This study does not involve or apply this indicator.

c QOL, Quality of Life in Alzheimer’s Disease; MHI, Mental Health Inventory-5; GDS, SF–Geriatric Depression Scale–Short Form; PSS, Perceived Stress Scale; SF-36v2, Short Form Health Survey 36, Version 2; PANAS, SF–Positive and Negative Affect Schedule–Short Form; URCS, Unidimensional Relationship Closeness Scale; RSS, Relationship Satisfaction Scale; CES-D, Center for Epidemiologic Studies Depression Scale; PSS, Perceived Stress Scale; SUS, System Usability Scale; PAS, Physical Activity Scale for the Elderly; PHQ-9, Patient Health Questionnaire-9; GAD-7, Generalized Anxiety Disorder-7; MOS-SS, Medical Outcomes Study-Sleep Scale; PROMIS-29, Patient-Reported Outcomes Measurement Information System-29; PSQI, Pittsburgh Sleep Quality Index; MoCA, Montreal Cognitive Assessment; CDR, Clinical Dementia Rating; GDS, Geriatric Depression Scale; RMBPC, Revised Memory and Behavior Problems Checklist; ZBI, Zarit Burden Interview; COPM, Canadian Occupational Performance Measure; NPI, Neuropsychiatric Inventory; SE, Sleep Efficiency; AMRT, Assessment of Motor and Respiratory Tasks; NPI Total Score, Neuropsychiatric Inventory Total Score; NPI Caregiver Distress, Neuropsychiatric Inventory Caregiver Distress; ASCO, Adult Social Care Outcomes Toolkit; MSPP, Multi-Step Physical Performance; PES, Participation Enjoyment Scale; SSCQ, Short Sense of Competence Questionnaire; TOPICS, MDS, The Older Persons and Informal Caregivers Survey–Minimum Data Set; QUALIDEM, Quality of Life in Dementia; ADQ, Approaches to Dementia Questionnaire; EDIZ, Caregiver Burden Scale (Dutch version); Carer Qol, Care-Related Quality of Life; CSI, Couple Satisfaction Index; PCS, Preparedness for Caregiving Scale; VC, IOE, Video Coding, Incorporating Observed Emotion scale; DAD, Disability Assessment for Dementia; SDI, Sleep Disturbance Index; QALYs, Quality-Adjusted Life Years; FAST, Functional Assessment Staging Tool; IADL, Instrumental Activities of Daily Living; MCTS, Modified Conflict Tactics Scale; ObsRvR, Observing Reactions in Virtual Reality; SPPB, Short Physical Performance Battery; Tech pH, Technology Philiac Questionnaire; RAND-36, RAND 36-Item Health Survey; Cantril**’**s Ladder, Cantril Self-Anchoring Scale; VAS, Visual Analogue Scale; WEMWBS (short form), Warwick-Edinburgh Mental Well-being Scale (short form); NPT Constructs, Normalization Process Theory Constructs; SA, Social Alarm; CAFU, Caregiver Assessment of Functioning and Upset; CMI, Care Management Inventory; SPPB, Short Physical Performance Battery; SMS, Stress Management Scale; RCSES, Revised Caregiving Self-Efficacy Scale.

Following full-text assessment, the MMAT tool was used to evaluate the quality of the 35 included studies. Among these, 19 studies were rated as Grade A (11, 23–25, 30, 33–36, 39, 40, 43–46, 51–53, 57), 8 studies were rated as Grade B (27–29, 41, 42, 48, 49, 56), and 8 studies were rated as Grade C (17, 18, 32, 37, 38, 47, 54, 55). Three studies were research protocols (26, 31, 50); as protocols are planning documents rather than outcome reports, they were not subject to quality assessment. Detailed evaluation processes and results are presented in **Supplementary Table 3**.

### 3.3 Intervention modalities

**Figure 2:**
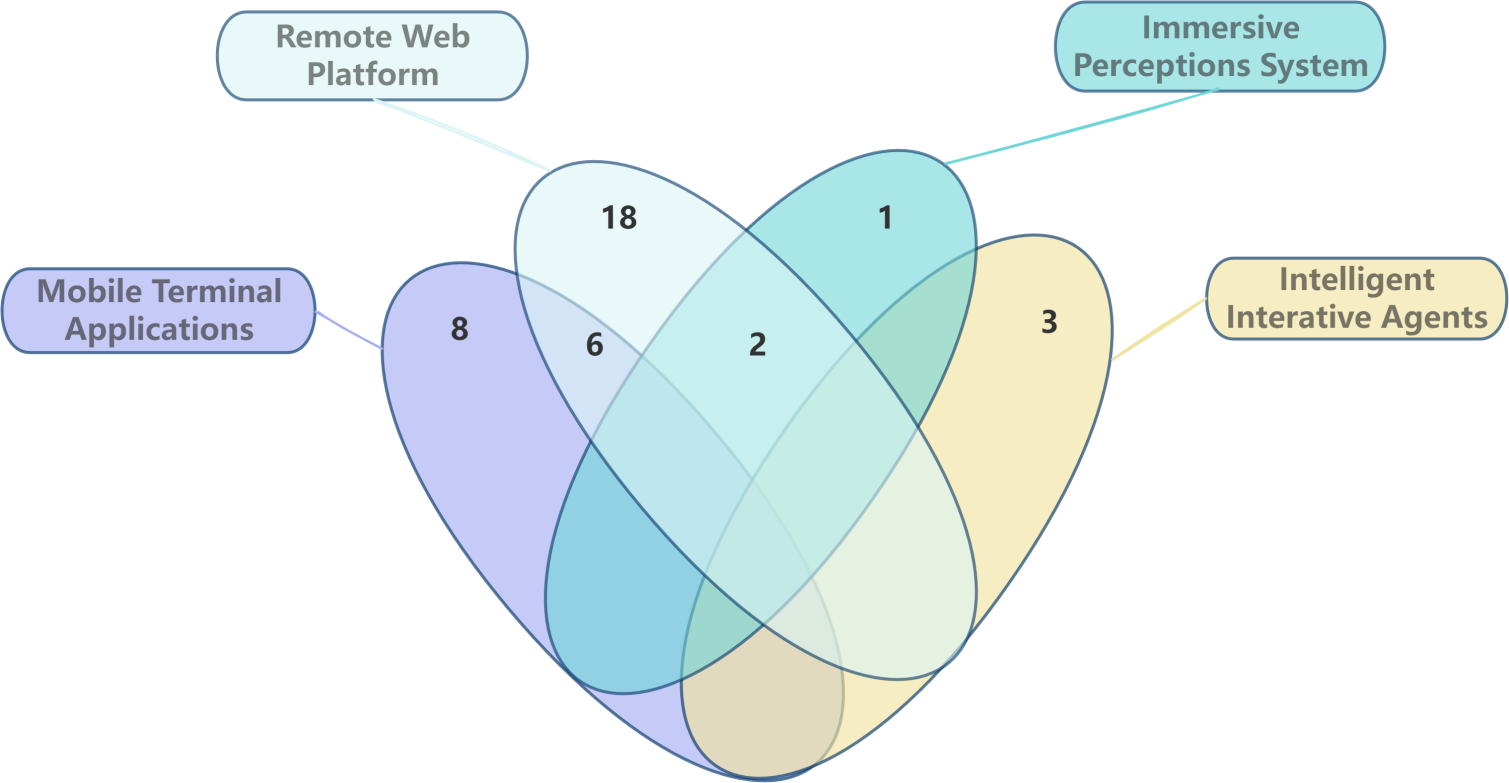
Type of digital technology. (18 used remote web platforms, 8 used mobile applications, 1 utilized immersive systems, 3 employed intelligent agents, 6 applied both mobile applications and web platforms, and 2 combined immersive systems with web platforms).

Among the included interventions, 35 interventions (11, 17, 18, 23–34, 36–44, 46–55, 57) required joint participation of patients and caregivers, while 3 interventions (35, 45, 56) were patient- or caregiver-led with the other party providing support. In terms of technical implementation, the interventions primarily relied on sensory immersive systems (11), (n=1), mobile applications (18, 24, 27, 30, 35, 44, 48, 53), (n=8), remote web platforms (23, 25, 26, 29, 31, 32, 34, 37–43, 46, 49, 51, 52), (n=18), and intelligent interactive agents (33, 54, 55), (n=3). Two studies combined sensory immersive systems with remote web platforms (28, 36), (n=2), concurrent use of mobile terminal applications and remote network platforms (17, 45, 47, 50, 56, 57), (n=6), as illustrated in **Figure 2**. Overall, remote interventions utilizing tablet applications alongside video and conferencing platforms are most prevalent, with interaction-based intervention designs demonstrating superior collaborative outcomes. Existing studies predominantly employ remote video communication and reminder functions to deliver psychoeducation and social support.

Sensory immersive technologies are often realized through virtual environment techniques and are generally classified into three levels of immersion. Low-immersion systems present simulated scenarios on electronic screens, enabling simple joint interactions between patients and caregivers; medium-immersion systems employ multi-screen or surround-projection displays to enhance audiovisual engagement (11); and high-immersion systems, or immersive virtual reality (IVR), utilize head-mounted displays and sensory devices to deliver fully multisensory environments (58). Existing research has explored these three categories of sensory-immersion applications. In Afifi et al.’s study (28), elderly participants used standalone head-mounted devices to engage in immersive scenario exercises, while family caregivers participated remotely. Results indicated that the intervention enhanced patients’ sense of social presence and promoted shared reminiscence among caregivers, although no significant improvement was observed in overall dyadic relationship quality. In Appel et al.’s study (36), individuals with dementia viewed 360-degree videos through VR headsets, while caregivers monitored their responses in real time via tablets to facilitate synchronized experiences. Another study employing low-immersion technology (11) used computer-generated natural soundscapes and virtual travel scenarios to facilitate shared participation, with content tailored to participants’ preferences and cognitive abilities. This approach was shown to alleviate patient loneliness and enhance caregiving experiences.

Compared to VR technology, tablet- or smartphone-based mobile applications have been more frequently used in home settings owing to their lower cost and wider accessibility (24, 56). Current interventions typically include cognitive training, self-management, symptom monitoring, and social interaction. Muñoz et al.’s study (48) reported that tablet-based applications incorporating competitive and cooperative games facilitated shared participation through nonverbal gameplay between patients and caregivers. This format allowed caregivers to more intuitively observe patients’ abilities and engagement levels, thereby improving their understanding of behavioral patterns. Other studies have promoted positive dyadic interactions through shared physical-exercise activities, contributing to improved relationship quality (24, 48). However, several usability limitations have been identified for mobile applications. User-anonymity protections often restrict the collection of usage data, making it difficult to accurately determine participation levels and intervention dosage (35). In terms of interactivity, the absence of real-time dialogue functions requires some older adults to replay prompts multiple times to fully understand the content (35). Application stability also affects the overall intervention process, as unexpected application crashes or forced updates may interrupt sessions or require restarting. Operational issues—such as battery failures, overly sensitive touch screens, or screen glare—are also commonly reported (44). Furthermore, most existing mobile applications are not specifically designed for individuals with cognitive impairments, limiting their ability to sustain engagement or adapt to fluctuating cognitive capacities (56).

Remote web platforms generally comprise video-conferencing systems and online educational portals and are primarily used for remote support and psychosocial interventions. They facilitate triadic interactions among patients, caregivers, and professionals, allowing patients to receive cognitive and emotional support at home while simultaneously providing guidance to caregivers (39, 51). Compared with telephone-based interventions, video-based communication overcomes spatial barriers and has been shown to yield higher adherence, greater engagement, better communication quality, and deeper interaction, while more effectively eliciting synchronous exchanges (29, 32). The flexibility of remote platforms enables intervention content to be tailored to different stages of disease progression. For example, individuals in early stages typically receive enhanced social-activity and resource-related information, whereas those in middle-to-late stages rely on simplified interactions to maintain basic communication (43). Furthermore, online platforms offer informational and social-support resources for community-dwelling individuals with mild-to-moderate dementia or cognitive impairment and their informal caregivers, helping both parties remain connected beyond daily care and expanding the scope of dyadic interactions (34). A range of technical and operational barriers has been reported, including poor audiovisual quality, frequent platform updates that compromise usability (32), caregiver dropout caused by login difficulties (37), and patient challenges arising from technological complexity or the absence of ongoing training (43). Additionally, some intervention protocols lack clearly defined follow-up procedures, leaving users uncertain about subsequent steps (52).

AI-powered interactive agents are primarily used to support daily care through voice-based interaction, reminder functions, and environmental monitoring, with three studies investigating this intervention format (33, 54, 55). Research indicates that these systems can provide cognitive support to patients (55) and identify and signal abnormalities to caregivers (54), thereby enhancing collaborative health management. Stara et al. (55) implemented a 4-week intervention using a tablet-based interactive agent, “Anne,” and results demonstrated significant improvements in patient–caregiver closeness, indicating enhanced relational connectedness. However, the study also reported technical limitations, including inadequate voice-recognition performance, which diminished usability and user trust and increased operational burden for both patients and caregivers. Amabili et al. (54) developed the eWare system, which integrates sensor-based monitoring with social-robot interactions to track and intervene in patients’ lifestyle patterns while delivering personalized reminders. Simultaneously, the system supports caregivers by facilitating burden management and enabling real-time monitoring, thereby establishing a collaborative care mechanism.

### 3.4 Characteristics of participants

Participants included in this review primarily comprised individuals with mild cognitive impairment or dementia and their informal caregivers. Informal caregivers were predominantly spouses or adult children of patients (11, 17, 18, 23–25, 28, 30–33, 35, 38–43, 45–49, 51–53, 55) (n=27), with a minority are other family members or close friends (26, 34, 36, 44, 57) (n=5). Some studies simply describe them as family members (27, 29, 37, 50, 54, 56) (n=6). In some studies, professional or semi-formal caregivers participated alongside informal caregivers in the intervention (18, 35, 37, 46, 48) (n=5). These formal caregivers included nursing home attendants, caregivers, and healthcare professionals, who primarily assumed guiding and supportive roles (18). Examples included assisting with baseline data collection, providing patient information, and facilitating patient and family caregiver engagement in the intervention (36). Based on the role configuration of the dyad in the intervention, three categories can be identified (1): Informal caregiver-oriented dyads, where the caregiver is the primary user and assumes the intervention execution role (2); Collaborative dyads, where the patient and caregiver jointly access the system and participate synergistically (3); Patient-oriented dyads, where the intervention focuses on the patient themselves, with the caregiver providing auxiliary support.

### 3.5 Outcome measurement

The primary outcomes for dyadic interventions in the included studies were measured across domains such as quality of life, psychological outcomes, relationship quality, physical functioning, caregiver burden, and technology-related indicators. Quality of life was the most frequently evaluated outcome (n=16) (17, 28, 29, 31, 32, 35–37, 39, 44, 50, 51, 53–56), assessed using instruments including EQ-5D-5L, QoL-AD, SF-36V2, Care QoL, and QUALIDEM. Psychological outcomes were assessed in 19 studies (n=19) (17, 27–29, 31, 32, 34, 36, 37, 39, 44, 45, 47, 49–51, 53, 55, 56) using tools such as SMS, WEMWBS, VAS, NPI-RMBPC, MoCA, CDR, GDS, RMBDC, GAD-7, CES-D, PSS, GDS-SF, MHI-5, and PANAS-SF. Relationship and interaction quality was evaluated in 8 studies (26, 28, 31, 32, 44, 48, 52, 53) using instruments including URCS, RSS, PPS, MPC-D, MSC-D, INTERACT, and CSI. Physical functioning was assessed in 9 studies (17, 39, 42, 45, 49–51, 53, 56) using measures such as SPPB, PSQI, SE, and IADL. Caregiver burden was assessed in 15 studies (17, 27–29, 34, 37, 39, 42, 47, 49–51, 53, 54, 56) commonly using ZBI, RMBPC, NPI Caregiver Distress, EDIZ, CAFU, PSS, MOS-S, and PCS. Technology-related outcomes were prominent, with feasibility evaluated in 22 studies (11, 17, 23, 26, 27, 29, 30, 32, 34–37, 39, 41–46, 52, 54, 55, 57), often assessed based on telemedicine session completion rates, retention rates, SUS scales, usage logs, and study-defined feasibility metrics (41, 48); Technology acceptance was assessed in 17 studies (26–28, 31, 33, 35, 36, 41–46, 48, 52, 56, 57), predominantly through questionnaires such as the Usefulness and Usability Questionnaire and through interviews guided by the Unified Theory of Acceptance and Use of Technology (41, 43, 55), User engagement and experience were documented in 8 studies (35, 41–43, 45, 46, 52, 57), involving interactions with applications, virtual reality systems, or social robots. Overall, the studies encompassed outcomes across both patient and caregiver levels, including quality of life, psychological status, relational dynamics, care experiences, and technology adaptability.

### 3.6 Summary of intervention outcomes

Digital technology–based dyadic interventions were reported to demonstrate positive outcomes in most studies. Among these, 3 studies reported statistically significant beneficial effects (30, 33, 34), whereas 5 reported no significant outcomes (25, 27, 29, 42, 55). Additionally, 14 studies reported mixed results, demonstrating both beneficial effects and non-significant differences (17, 18, 28, 37–39, 44, 45, 47, 49, 51, 53, 54, 56). These mixed outcomes often arose from comprehensive evaluations of clinical efficacy, including caregiver burden, quality of life, and cognitive function, as well as technology acceptance, trust, and relationship quality. Studies with beneficial outcomes showed effect sizes that were predominantly in the moderate-to-high range (29, 39, 47, 51). 6 studies indicated that patient improvements were primarily observed in cognitive function, emotional regulation, and social interaction (27, 28, 32, 36, 51, 54), whereas 8 studies reported reductions in behavioral and psychological symptoms (17, 29, 37, 39, 42, 47, 49, 55). 6 studies also documented caregiver benefits, including reduced psychological burden, enhanced self-efficacy, and increased caregiving confidence (29, 37, 39, 42, 47, 49). Furthermore, the available evidence predominantly relies on short-term follow-up assessments, capturing only immediate post-intervention improvements; long-term effects remain insufficiently examined and require further verification.

### 3.7 Facilitating and barrier factors

Among the included studies, facilitating and hindering factors related to the implementation of digital dyadic interventions for patients with mild cognitive impairment or dementia and their caregivers were summarized across three dimensions: user engagement, technology and resource constraints, and regional economic conditions. Regarding user engagement, 14 studies reported that cognitive decline, sensory deficits, and attention impairments in patients were found to affect task comprehension and device operation (17, 23, 28, 30, 32, 33, 35, 44–46, 48, 49, 56, 57), the degree of cognitive decline, sensory deficits, and attention impairments in patients were reported to affect task comprehension and device operation (56, 59).Technical proficiency challenges were reported among spousal caregivers of older adults, and disparities in caregivers’ digital skills were found to influence mutual engagement and the quality of interactions (n=9) (32–36, 40, 43, 52, 53). Economic status was examined in 2 studies (28, 57), while distance from patients and relational closeness were also identified as critical factors affecting caregiver participation (25, 34, 35). The impact of internet connectivity was examined in 5 studies (23, 28, 29, 34, 41). Inadequate connectivity was commonly reported among individuals living alone or in rural households (28), thereby compromising the stability of remote interventions. The high cost of acquiring and maintaining equipment, together with frequent platform updates, was reported to increase resource burden (32, 45). A total of five studies also reported that privacy and data-security concerns adversely affected participant engagement (17, 33, 34, 42, 57). Geographically and economically, existing literature was predominantly derived from high-income regions such as North America, Europe, and Oceania, where healthcare infrastructures and digital-health ecosystems are more mature. As a result, the available evidence is skewed toward developed countries, which may limit its applicability in low- and middle-income regions. Additionally, most studies were still in developmental or pilot phases, thereby contributing to uncertainty about intervention effectiveness and scalability.

## 4 Discussion

This scoping review evaluated the current application of digital-based dyadic interventions for individuals with MCI or dementia and their caregivers. The findings indicated that these interventions encompassed diverse formats and demonstrated high overall feasibility and acceptability. These interventions were found to improve patients’ cognitive and psychosocial functioning while enhancing caregivers’ disease understanding and coping abilities, thereby strengthening mutual interaction and emotional bonds. Furthermore, studies have revealed (60) that when technology was applied solely to patients, discontinuation or insufficient engagement frequently occurred due to cognitive impairment and operational difficulties. In contrast, the collaborative dual-participation model was shown to more effectively facilitate consensus building, supportive relationship establishment, and improvements in quality of life and relationship satisfaction through interaction (18). Therefore, building upon the above foundations, this study will examine different types of digital intervention technologies while exploring their mechanisms of action, cross-cultural implementation, and practical implications.

### 4.1 Sensory immersive technologies

Sensory immersive technologies are considered to offer shared immersive experiences in dyadic interventions for individuals with cognitive impairment, although the existing evidence base remains limited and largely exploratory. Current findings indicate that VR was not associated with significant improvements in family relationship quality in the study by Afifi et al. (28). This outcome may be attributable to participants’ strong baseline relationships and higher socioeconomic status, indicating that future samples should include more socioeconomically diverse populations to strengthen external validity. Furthermore, to accommodate users with varying cognitive abilities, system design should integrate multiple interaction modalities, including voice-command functions (11). Currently, the adoption of sensory-immersion systems remains limited by network conditions, equipment costs, and operational complexity. In addition, head-mounted IVR devices may pose safety risks such as motion sickness and falls (36), while the associated sense of isolation may reduce natural caregiver–patient interaction. High-quality VR video has also been shown to affect users’ long-term willingness to engage with dual-device systems (36), yet immersive content specifically tailored to individuals with cognitive impairment remains limited. Therefore, future research should prioritize the optimization of both immersive content and device design, including the development of more personalized, high-quality content and improvements in device portability and safety. These advancements would facilitate the stable implementation and long-term adoption of immersive systems in home and community settings.

### 4.2 Mobile applications

Mobile applications for caregiving are designed with intuitive interfaces and instructional guidance, enabling caregivers to support patients while acquiring care-related knowledge under professional oversight (18). This approach has been shown to enhance patient independence and strengthen caregivers’ competence and confidence. Existing research indicates that although mobile apps are widely accessible in home environments, their effectiveness is shaped by device stability, functional adaptability, and patients’ motivation to engage. Future research should prioritize improvements in system stability and personalized adaptability by optimizing app design (53), streamlining interaction processes, and ensuring sustained technical support. This approach is better suited to the evolving functional capacities of individuals with cognitive impairment and their caregivers. Additionally, more secure data-collection strategies should be implemented to ensure full protection of privacy, such as capturing only anonymized usage frequency and interaction patterns. Simultaneously, the integration of wearable devices or other biofeedback technologies could facilitate the dynamic assessment of collaborative engagement between patients and caregivers, thereby supporting improvements in intervention effectiveness and scalability (27, 48).

### 4.3 Remote online platforms

Remote online platforms have been shown to offer advantages in delivering cognitive support, facilitating family communication, and strengthening caregiver capabilities. Their tripartite interaction model is considered to extend professional support into the home environment, helping to maintain cognitive function, enhance intrafamilial communication, and improve caregivers’ comprehension and application of health-related information, thereby facilitating more effective emotional co-regulation. Research has indicated that internet-based co-creation or content-sharing activities may enable caregivers to gain deeper insight into patients’ identities and emotional experiences, thereby enhancing empathic understanding and strengthening relationship quality (17). However, existing research remains predominantly developmental, and practical outcomes are largely contingent on technological infrastructure and platform design. Current platforms do not incorporate operational logic specifically tailored to individuals with cognitive impairment, and both patients and caregivers often lack sustained and accessible technical support. Moreover, individuals with declining cognitive abilities incur greater cognitive and practical demands when attempting to learn new technologies. Without clear, step-by-step guidance, discontinuation of use is more likely to occur. Therefore, future platform designs should prioritize optimizing user experience by minimizing operational steps, incorporating more structured navigation prompts, and ensuring continuous technical assistance. This includes providing clear, manualized intervention procedures (39) to improve procedural coherence and strengthen task-completion feedback mechanisms.

### 4.4 AI interaction agents

Artificial intelligence (AI) interactive agents constitute a class of digital technologies or physical entities capable of interacting with humans via voice and other linguistic modalities. These agents can provide information, execute tasks, or serve as companions (55). As an emerging form of digital intervention, AI interactive agents are increasingly applied to assist in the daily care of individuals with cognitive impairment and their informal caregivers. Currently, these interventions remain exploratory, as most studies feature small sample sizes and predominantly quasi-experimental or cohort designs, lacking large-scale randomized controlled trials. Their effectiveness requires further validation. Furthermore, the effectiveness of these interventions depends on caregivers’ initial parameter configuration and guidance (33). When caregivers face substantial care burdens, their limited availability may hinder proper system use and undermine the intended effectiveness of the intervention. Patients may also experience reduced engagement as cognitive abilities decline, thereby limiting intervention continuity. Technical performance substantially influences user experience, and existing studies indicate that unstable voice-recognition functions disrupt interaction flow and diminish system usability and perceived trustworthiness (55). Future research should include validation through larger-scale studies and prioritize system enhancements, including greater personalization and simplified operational workflows. Adaptability for users with differing cognitive abilities should be strengthened to reduce operational burden. Concurrently, attention should be directed toward how contextual factors—such as residential distance, family structure, and emotional closeness—shape user experience, in order to identify optimal applications for alleviating caregiver isolation and fostering stronger family connections.

### 4.5 Mechanisms of action and synergistic effects of dyadic interventions

The mechanism of action of Dyadic interventions can be classified into two primary pathways (1): patient-oriented and (2) caregiver-oriented approaches. Patients are engaged in regular cognitive training through video conferencing, mobile applications, or virtual reality to support cognitive functioning and independence. Caregivers are provided with educational resources and psychological support through remote platforms, which help alleviate burden, improve emotional well-being, and enhance caregiving competence. Second, Dyadic interventions directly affect the dyadic relationship by promoting cooperation and intimacy through emotional co-regulation, shared goal-setting, and synchronous interaction (25), thereby enhancing overall relationship quality Overall, the value of Dyadic interventions is reflected in the transformation of individual improvements into relational synergies through reciprocal interaction. Preserving patients’ cognitive and emotional well-being helps to alleviate caregiver stress, while improvements in caregivers’ competence and psychological health, in turn, foster patient engagement. This ultimately establishes a virtuous cycle that strengthens mutual understanding and support, thereby enhancing the overall effectiveness of the intervention. Future intervention designs should consider incorporating technological support and caregiver assistance to facilitate patient expression and participation, while accounting for individual capacity differences, thereby preserving the bidirectional nature of interaction to the greatest extent possible.

### 4.6 Cross-cultural implementation and technology accessibility

Existing research indicates that the implementation status of digital dual interventions varies among countries with varying levels of economic development. In high-income countries, including the United States, the United Kingdom, Canada, the Netherlands, Norway, Italy, and Sweden, well-established healthcare systems and widespread adoption of digital devices provide favorable conditions for the implementation of digital dual interventions. However, research data indicate that 68% of dementia cases are reported in low- and middle-income countries (2), highlighting a mismatch between intervention needs and resource availability. Some high-income countries also exhibit the phenomenon of “high installation rates but low actual usage” (45), suggesting that hardware accessibility does not necessarily translate into effective utilization. Cost burdens, privacy and ethical concerns, and the lack of sustained technical support can all undermine the effectiveness of interventions. In contrast, research conducted in developing countries, such as China, tends to favor lower-cost and more widely accessible mobile applications, including collaborative care systems based on WeChat mini-programs (50), to address rapidly increasing care demands and resource constraints in the context of an aging population. Cultural contexts are also known to influence technology acceptance and usage patterns. Cultural contexts are also known to influence technology acceptance and usage patterns. Research conducted by Valdivia et al. (35) indicates that, within South Asian cultures characterized by multigenerational households and strong familial obligations, caregiving is frequently regarded as an extension of emotional duty. Families in these cultures may approach external technological assistance with increased caution, thereby reducing proactive adoption. Conversely, in cultures that emphasize individual autonomy, digital technologies are more readily perceived as tools to enhance personal independence and to alleviate caregiving burdens. Furthermore, cross-national comparisons have highlighted variations in digital literacy across different cultural contexts. For instance, significant differences in technological proficiency have been observed among patients with mild cognitive impairment in Sweden and Spain (56). These disparities suggest that identical interventions may result in distinct usage behaviors, levels of dependency, and technical support requirements across different countries. Consequently, the design and implementation of digital interventions must comprehensively consider local cultural backgrounds, family structures, and healthcare infrastructures to enhance both acceptability and sustained utilization.

### 4.7 Implications for future research

Future research should prioritize the enhancement of personalization and user experience in digital dual interventions, the improvement of accessibility and sustainability of digital technology utilization, and the further reduction of cognitive load for individuals with cognitive impairment during technology interaction. This necessitates a more in-depth investigation into how technology can be integrated into individuals’ daily lives and experiential practices (57). Building upon this foundation, further research may investigate the role of digital health technologies, including immersive virtual environments and voice reminders, that enhance emotional well-being, communication, and relationship quality among patients and caregivers. The efficacy of combining technological interventions with non-pharmacological measures, such as sleep education, mindfulness training, or home-based exercise, should also be examined. Additionally, communication and emotional management training for patients and caregivers should be strengthened to enhance dyadic resilience in coping with caregiving stress and evolving demands. Enhancement of cybersecurity awareness and privacy protection capabilities among patients and caregivers is equally essential. Future research should investigate combined security mechanisms—such as message verification codes and biometric authentication—to reinforce system security. Concurrently, cybersecurity training should be provided to equip users with the ability to identify fraud risks and appropriately configure privacy settings, thereby minimizing information leakage and fostering trust.

Overall, the enhancement of accessibility and sustainability requires continuous improvements in user training, technology adaptation, network infrastructure, and privacy protection. Simultaneously, incorporating sensory adaptation and contextual customization into design, which provides personalized support based on individuals’ economic status, relationship type, living conditions, cultural background, and levels of technological proficiency as reported in prior studies (57) facilitates more effective interactions between dyads with differing cognitive abilities. This approach ultimately enhances compliance and the effectiveness of interventions. Future efforts should aim to establish more standardized and inclusive implementation approaches that accommodate diverse cultural and economic contexts, as highlighted in previous research.

## 5 Limitations

This study has certain limitations. First, the included studies varied considerably in technology type, intervention content, implementation frequency, and follow-up duration, with no uniform standards. These variations hindered the integration and comparability of findings and may have affected the overall conclusions drawn from this review. Most studies reported only short-term outcomes, making it difficult to demonstrate the sustained effects of digital dual interventions in long-term care. Intervention effectiveness is influenced by participants’ digital literacy. Elderly patients and caregivers often encounter difficulties operating digital devices, which may lead to accessibility gaps and introduce sampling bias. Furthermore, the majority of included studies originated from high-income regions, including North America, Europe, and Oceania, which limits the applicability of the conclusions to low- and middle-income settings.

Second, methodological limitations are inherent to this review. The included studies may have been affected by publication availability, as some existed only as preprints, dissertations, or institutional reports rather than formally published articles. Future research should enhance the robustness and generalizability of findings by employing standardized study designs, conducting long-term follow-up, providing skill training, and expanding literature sources.

## 6 Conclusions

The reviewed studies suggest that digital dyadic interventions are effective in enhancing quality of life, cognitive function, and emotional well-being among individuals with MCI or dementia, as well as their caregivers. They also show distinct advantages in reducing caregiver burden and fostering relational interactions. However, limited technological accessibility, disparities in user proficiency, and inconsistent participation in dyadic interaction models remain major challenges influencing their implementation and effectiveness. Overall, digital dyadic interventions represent an innovative and potentially sustainable approach to dementia care, with promising applications in home and community settings that warrant further investigation and rigorous validation.

## Data Availability

The original contributions presented in the study are included in the article/**Supplementary Material**. Further inquiries can be directed to the corresponding author.

## References

[1] Hao M Chen J . Trend analysis and future predictions of global burden of alzheimer’s disease and other dementias: a study based on the global burden of disease database from 1990 to 2021. BMC Med. (2025) 23:378. doi: 10.1186/s12916-025-04169-w, PMID: 40597083 PMC12220445

[2] Jia L Du Y Chu L Zhang Z Li F Lyu D . Prevalence, risk factors, and management of dementia and mild cognitive impairment in adults aged 60 years or older in China: a cross-sectional study. Lancet Public Health. (2020) 5:e661–71. doi: 10.1016/S2468-2667(20)30185-7, PMID: 33271079

[3] Petersen RC Doody R Kurz A Mohs RC Morris JC Rabins PV . Current concepts in mild cognitive impairment. Arch Neurol. (2001) 58:1985. doi: 10.1001/archneur.58.12.1985, PMID: 11735772

[4] Petersen RC Lopez O Armstrong MJ Getchius TSD Ganguli M Gloss D . Practice guideline update summary: Mild cognitive impairment [RETIRED]: Report of the Guideline Development, Dissemination, and Implementation Subcommittee of the American Academy of Neurology. Neurology. (2018) 90:126–35. doi: 10.1212/WNL.0000000000004826, PMID: 29282327 PMC5772157

[5] Hugo J Ganguli M . Dementia and cognitive impairment: epidemiology, diagnosis, and treatment. Clin Geriatr Med. (2014) 30:421–42. doi: 10.1016/j.cger.2014.04.001, PMID: 25037289 PMC4104432

[6] 2024 Alzheimer’s disease facts and figures. Alzheimers Dement. (2024) 20:3708–821. doi: 10.1002/alz.13809, PMID: 38689398 PMC11095490

[7] International AD Patterson C . World Alzheimer Report 2018: The state of the art of dementia research: New frontiers (2018). Available online at: https://www.alzint.org/resource/world-alzheimer-report-2018/ (Accessed July 23, 2025).

[8] Richardson TJ Lee SJ Berg-Weger M Grossberg GT . Caregiver health: health of caregivers of alzheimer’s and other dementia patients. Curr Psychiatry Rep. (2013) 15:367. doi: 10.1007/s11920-013-0367-2, PMID: 23712718

[9] Sun V Raz DJ Kim JY . Caring for the informal cancer caregiver. Curr Opin Support Palliat Care. (2019) 13:238. doi: 10.1097/SPC.0000000000000438, PMID: 31157656 PMC6669089

[10] Paradise M McCade D Hickie IB Diamond K Lewis SJG Naismith SL . Caregiver burden in mild cognitive impairment. Aging Ment Health. (2015) 19:72–8. doi: 10.1080/13607863.2014.915922, PMID: 24866046

[11] Rochon EA Thacker A Phillips M Ritchie C Vranceanu AM Plys E . Developing a dyadic immersive virtual environment technology intervention for persons living with dementia and their caregivers: multiphasic user-centered design study. JMIR AGING. (2025) 8:e66212. doi: 10.2196/66212, PMID: 40397932 PMC12138290

[12] Pucciarelli G Lommi M Magwood GS Simeone S Colaceci S Vellone E . Effectiveness of dyadic interventions to improve stroke patient-caregiver dyads’ outcomes after discharge: A systematic review and meta-analysis study. Eur J Cardiovasc Nurs. (2021) 20:14–33. doi: 10.1177/1474515120926069, PMID: 33570593

[13] Wang X Zang L Hui X Meng X Qiao S Fan L . Dyadic interventions for cancer patient-caregiver dyads: A systematic review and network meta-analysis. Int J Nurs Stud. (2025) 161:104948. doi: 10.1016/j.ijnurstu.2024.104948, PMID: 39566302

[14] Poon E . A systematic review and meta-analysis of dyadic psychological interventions for BPSD, quality of life and/or caregiver burden in dementia or MCI. Clin Gerontol. (2022) 45:777–97. doi: 10.1080/07317115.2019.1694117, PMID: 31752633

[15] Mittermaier M Venkatesh KP Kvedar JC . Digital health technology in clinical trials. NPJ Digit Med. (2023) 6:88. doi: 10.1038/s41746-023-00841-8, PMID: 37202443 PMC10195788

[16] Cure P Radman T Doyle JM Atienza AA Fessel JP Hartshorn CM . Digital health technology research funded by the national institutes of health. JAMA Netw Open. (2025) 8:e2452976. doi: 10.1001/jamanetworkopen.2024.52976, PMID: 39752153

[17] Elfrink TR Ullrich C Kunz M Zuidema SU Westerhof GJ . The Online Life Story Book: A randomized controlled trial on the effects of a digital reminiscence intervention for people with (very) mild dementia and their informal caregivers. PloS One. (2021) 16:e0256251. doi: 10.1371/journal.pone.0256251, PMID: 34525105 PMC8443059

[18] Beentjes KM Neal DP Kerkhof YJF Broeder C Moeridjan ZDJ Ettema TP . Impact of the FindMyApps program on people with mild cognitive impairment or dementia and their caregivers; an exploratory pilot randomised controlled trial. Disabil Rehabil Assist Technol. (2023) 18:253–65. doi: 10.1080/17483107.2020.1842918, PMID: 33245000

[19] Arksey H O’Malley L . Scoping studies: towards a methodological framework. Int J Soc Res Methodol. (2005) 8:19–32. doi: 10.1080/1364557032000119616, PMID: 39989647

[20] Page MJ McKenzie JE Bossuyt PM Boutron I Hoffmann TC Mulrow CD . The PRISMA 2020 statement: an updated guideline for reporting systematic reviews. Syst Rev. (2021) 10:89. doi: 10.1186/s13643-021-01626-4, PMID: 33781348 PMC8008539

[21] Peters MDJ Marnie C Tricco AC Pollock D Munn Z Alexander L . Updated methodological guidance for the conduct of scoping reviews. JBI Evid Synth. (2020) 18:2119. doi: 10.11124/JBIES-20-00167, PMID: 33038124

[22] Hong QN Fàbregues S Bartlett G Boardman F Cargo M Dagenais P . The Mixed Methods Appraisal Tool (MMAT) version 2018 for information professionals and researchers. Educ Inf. (2018) 34:285–91. doi: 10.3233/EFI-180221, PMID: 39743787

[23] Bannon SM McCage S Walker K Brewer J Ahmad N Cornelius T . Resilient together for dementia: A qualitative study of couples’ treatment preferences to address distress early after diagnosis. J Alzheimers Dis JAD. (2025) 105:808–824. doi: 10.1177/13872877251332658, PMID: 40261286 PMC12281600

[24] Kuzmik A Rodriguez M Hannan J Boltz M . Designing a mobile application to promote physical activity in spousal care partners of persons living with dementia and their care-recipient. Dement-Int J Soc Res Pract. (2025) 24:408–23. doi: 10.1177/14713012241272878, PMID: 39102469 PMC11915756

[25] Song Y Papazyan A Lee D Mitchell MN McCurry SM Irwin MR . The feasibility of a sleep education program for informal dementia care dyads: A pilot randomized controlled trial. J Am Geriatr Soc. (2024) 72:1207–15. doi: 10.1111/jgs.18720, PMID: 38193336 PMC11018508

[26] Rochon EA Sy M Phillips M Anderson E Plys E Ritchie C . Bio-experiential technology to support persons with dementia and care partners at home (TEND): protocol for an intervention development study. JMIR Res Protoc. (2023) 12:e52799. doi: 10.2196/52799, PMID: 38157239 PMC10787328

[27] Rodriguez MJ Kercher VM Jordan EJ Savoy A Hill JR Werner N . Technology caregiver intervention for Alzheimer’s disease (I-CARE): Feasibility and preliminary efficacy of Brain CareNotes. J Am Geriatr Soc. (2023) 71:3836-3847. doi: 10.1111/jgs.18591, PMID: 37706540 PMC10841172

[28] Afifi T Collins N Rand K Otmar C Mazur A Dunbar NE . Using Virtual Reality to Improve the Quality of Life of Older Adults with Cognitive Impairments and their Family Members who Live at a Distance. Health Commun. (2023) 38:1904–15. doi: 10.1080/10410236.2022.2040170, PMID: 35253531

[29] Rhodus EK Baum C Kryscio R Liu C George R Thompson M . Feasibility of telehealth occupational therapy for behavioral symptoms of adults with dementia: randomized controlled trial. Am J Occup Ther Off Publ Am Occup Ther Assoc. (2023) 77:7704205010. doi: 10.5014/ajot.2023.050124, PMID: 37624998 PMC10494967

[30] Peterson CM Birkeland RW Louwagie KW Ingvalson SN Mitchell LL Scott TL . Refining a driving retirement program for persons with dementia and their care partners: A mixed methods evaluation of carFreeMe™-dementia. J Gerontol B Psychol Sci Soc Sci. (2023) 78:506–519. doi: 10.1093/geronb/gbac151, PMID: 36149829 PMC9985324

[31] Bannon S Brewer J Ahmad N Cornelius T Jackson J Parker RA . A live video dyadic resiliency intervention to prevent chronic emotional distress early after dementia diagnoses: protocol for a dyadic mixed methods study. JMIR Res Protoc. (2023) 12:e45532. doi: 10.2196/45532, PMID: 37728979 PMC10551792

[32] Hastings SN Mahanna EP Berkowitz TSZ Smith VA Choate AL Hughes JM . Video-enhanced care management for medically complex older adults with cognitive impairment. J Am Geriatr Soc. (2021) 69:77–84. doi: 10.1111/jgs.16819, PMID: 32966603 PMC8579876

[33] Zubatiy T Vickers KL Mathur N Mynatt ED . (2021). Empowering dyads of older adults with mild cognitive impairment and their care partners using conversational agents, in: chi ‘21: proceedings of the 2021 chi conference on human factors in computing systems, New York, NY, USA: ACM (Association for Computing Machinery). doi: 10.1145/3411764.3445124, PMID:

[34] Peterson CM Mikal JP McCarron HR Finlay JM Mitchell LL Gaugler JE . The feasibility and utility of a personal health record for persons with dementia and their family caregivers for web-based care coordination: mixed methods study. JMIR AGING. (2020) 3:e17769. doi: 10.2196/17769, PMID: 32589158 PMC7381256

[35] Valdivia KP Li J . Voice familiarity in a voice-reminders app for elderly care recipients and their family caregivers. IEEE Trans Hum-Mach Syst. (2025) 55:529–538. doi: 10.1109/THMS.2025.3582259, PMID: 41116384

[36] Appel L Saryazdi R Lewis-Fung S Qi D Tesfaye EM Garito I . (2023). VRx@Home Pilot: Can Virtual Reality Therapy Improve Quality of Life for People with Dementia Living at Home?, In: Proceedings of the 2023 IEEE International Conference on Systems, Man, and Cybernetics (SMC). New York, NY, USA: IEEE. pp. 4542–9. Available online at: https://ieeexplore.ieee.org/document/10394094/ (Accessed July 27, 2025).

[37] Dröes RM van Rijn A Rus E Dacier S Meiland F . Utilization, effect, and benefit of the individualized Meeting Centers Support Program for people with dementia and caregivers. Clin Interv Aging. (2019) 14:1527–1553. doi: 10.2147/CIA.S212852, PMID: 31692559 PMC6717152

[38] Gonzalez L Rapaport P Livingston G Amador S Adeleke MO Barber JA . Cost–utility analysis of the DREAMS START intervention for people living with dementia and their carers: a within-trial economic evaluation. Lancet Healthy Longev. (2025) 6:100708. doi: 10.1016/j.lanhl.2025.100708, PMID: 40412418

[39] Cooper C Vickerstaff V Barber J Phillips R Ogden M Walters K . A psychosocial goal-setting and manualised support intervention for independence in dementia (NIDUS-Family) versus goal setting and routine care: a single-masked, phase 3, superiority, randomised controlled trial. Lancet Healthy Longev. (2024) 5:e141–51. doi: 10.1016/S2666-7568(23)00262-3, PMID: 38310894 PMC10834374

[40] Wolverson E White C Dunn R Cunnah K Howe D Paulson K . The use of a bespoke website developed for people with dementia and carers: Users’ experiences, perceptions and support needs. Dement Int J Soc Res Pract. (2022) 21:94–113. doi: 10.1177/14713012211028495, PMID: 34187203

[41] Howe D Thorpe J Dunn R White C Cunnah K Platt R . The CAREGIVERSPRO-MMD platform as an online informational and social support tool for people living with memory problems and their carers: an evaluation of user engagement, usability and usefulness. J Appl Gerontol Off J South Gerontol Soc. (2020) 39:1–10. doi: 10.1177/0733464819885326, PMID: 31690170

[42] Fowler-Davis S Barnett D Kelley J Curtis D . Potential for digital monitoring to enhance wellbeing at home for people with mild dementia and their family carers. J Alzheimers Dis JAD. (2020) 73:867–872. doi: 10.3233/JAD-190844, PMID: 31884471 PMC7081090

[43] Killin LOJ Russ TC Surdhar SK Yoon Y McKinstry B Gibson G . Digital Support Platform: a qualitative research study investigating the feasibility of an internet-based, postdiagnostic support platform for families living with dementia. BMJ Open. (2018) 8:e020281. doi: 10.1136/bmjopen-2017-020281, PMID: 29654028 PMC5898353

[44] Tyack C Camic Pm . Viewing art on a tablet computer: A well-being intervention for people with dementia and their caregivers. J Appl Gerontol Off J South Gerontol Soc. (2017) 36:1–41. doi: 10.1177/0733464815617287, PMID: 26675353

[45] Puaschitz NGS Jacobsen FF Berge LI Husebo BS . Access to, use of, and experiences with social alarms in home-living people with dementia: Results from the LIVE@Home. Path trial. Front Aging Neurosci. (2023) 15. doi: 10.3389/fnagi.2023.1167616, PMID: 37284020 PMC10239917

[46] Clark I Christopher N Stretton-Smith P Lawson K . The experiences of people living with dementia and their care partners participating in an online therapeutic songwriting program. Dement Lond Engl. (2024) 23:251–271. doi: 10.1177/14713012231224069, PMID: 38131325 PMC10807188

[47] Lang T Daniel K Inskip M Mavros Y Fiatarone Singh Am MA . Caring for informal dementia caregivers and their loved ones via the HOMeCARE exercise and mindfulness for health program (HOMeCARE): A randomized, single-blind, controlled trial. Gerontol Geriatr Med. (2023) 9:1–12. doi: 10.1177/23337214231203472, PMID: 37811133 PMC10559724

[48] Muñoz D Favilla S Pedell S Murphy A Beh J Petrovich T . (2021). Evaluating an app to promote a better visit through shared activities for people living with dementia and their families, in: Proceedings of the 2021 CHI Conference on Human Factors in Computing Systems, Yokohama Japan. pp. 1–13. New York, NY, USA: ACM (Association for Computing Machinery). doi: 10.1145/3411764.3445764, PMID:

[49] Laver K Liu E Clemson L Davies O Gray L Gitlin LN . Does telehealth delivery of a dyadic dementia care program provide a noninferior alternative to face-to-face delivery of the same program? A randomized, controlled trial. Am J Geriatr Psychiatry Off J Am Assoc Geriatr Psychiatry. (2020) 28:673–683. doi: 10.1016/j.jagp.2020.02.009, PMID: 32234275

[50] Wan Y Cai Y Liao S Zhao Q Wang Y Song X . Smartphone-based versus traditional face-to-face collaborative care for community-dwelling older adults living with dementia in China: protocol for an implementation science-based sequential multiple assignment randomised trial. BMJ Open. (2023) 13:e067406. doi: 10.1136/bmjopen-2022-067406, PMID: 37423624 PMC10335512

[51] Lai FH Yan EW Yu KK Tsui WS Chan DT Yee BK . The protective impact of telemedicine on persons with dementia and their caregivers during the COVID-19 pandemic. Am J Geriatr Psychiatry. (2020) 28:1175–84. doi: 10.1016/j.jagp.2020.07.019, PMID: 32873496 PMC7413846

[52] Monnet F Pivodic L Dupont C Smets T De Vleminck A Van Audenhove C . Evaluation of interactive web-based tools to stimulate reflection and communication about advance care planning with people with dementia and their family caregivers. BMC Palliat Care. (2024) 23:162. doi: 10.1186/s12904-024-01486-4, PMID: 38943119 PMC11212172

[53] Hoel V Ambugo EA Wolf-Ostermann K . Sustaining our relationship: dyadic interactions supported by technology for people with dementia and their informal caregivers. Int J Environ Res Public Health. (2022) 19:10956. doi: 10.3390/ijerph191710956, PMID: 36078671 PMC9518490

[54] Amabili G Cucchieri G Margaritini A Benadduci M Barbarossa F Luzi R . Social robotics and dementia: results from the eWare project in supporting older people and their informal caregivers. Int J Environ Res Public Health. (2022) 19:13334. doi: 10.3390/ijerph192013334, PMID: 36293915 PMC9603054

[55] Stara V Vera B Bolliger D Rossi L Felici E Di Rosa M . Usability and acceptance of the embodied conversational agent anne by people with dementia and their caregivers: exploratory study in home environment settings. JMIR MHealth UHealth. (2021) 9:e25891. doi: 10.2196/25891, PMID: 34170256 PMC8386369

[56] GP J BF P GP G A P JF L VM E . Attitudes and use of information and communication technologies in older adults with mild cognitive impairment or early stages of dementia and their caregivers: cross-sectional study. J Med Internet Res. (2020) 22:e17253. doi: 10.2196/17253, PMID: 32442136 PMC7296403

[57] Notley N Worthy P Peters ME Shaw J Nelson A Frost D . Encountering technology: A qualitative exploration of the technology experiences of people living with dementia and their care partners to inform knowledge, practice and technology design. Int Rev Psychiatry. (2025). doi: 10.1080/09540261.2025.2533898, PMID: 41799851

[58] Li Y Deng K . Application of virtual reality technology in the health field based on the background of big data. J Phys Conf Ser. (2021) 1883:012175. doi: 10.1088/1742-6596/1883/1/012175

[59] Ye M Liu Z Xie W Shou M Wang S Lin X . Implementation of telemedicine for patients with dementia and their caregivers: scoping review. J Med Internet Res. (2025) 27:e65667. doi: 10.2196/65667, PMID: 40324768 PMC12089872

[60] Starkhammar S Nygård L . Using a timer device for the stove: Experiences of older adults with memory impairment or dementia and their families. Technol Disabil. (2008) 20:179–91. doi: 10.3233/TAD-2008-20302, PMID: 39743787

